# High sensitivity detection of *Plasmodium *species reveals positive correlations between infections of different species, shifts in age distribution and reduced local variation in Papua New Guinea

**DOI:** 10.1186/1475-2875-8-41

**Published:** 2009-03-11

**Authors:** Ivo Mueller, Simone Widmer, Daniela Michel, Seri Maraga, David T McNamara, Benson Kiniboro, Albert Sie, Thomas A Smith, Peter A Zimmerman

**Affiliations:** 1 Papua New Guinea Institute of Medical Research, Goroka, PO Box 60, EHP 441, Papua New Guinea; 2 Swiss Tropical Institute, Department of Medical Parasitology and Infection Biology, and Department of Public Health and Epidemiology, Socinstrasse 57, PO Box, CH-4002 Basel, Switzerland; 3 Center for Global Health & Diseases, Case Western Reserve University, School of Medicine, Wolstein Research Building, 10900 Euclid Avenue, Cleveland, Ohio, 44106-7286, USA

## Abstract

**Background:**

When diagnosed by standard light microscopy (LM), malaria prevalence can vary significantly between sites, even at local scale, and mixed species infections are consistently less common than expect in areas co-endemic for *Plasmodium falciparum*, *Plasmodium vivax *and *Plasmodium malariae*. The development of a high-throughput molecular species diagnostic assay now enables routine PCR-based surveillance of malaria infections in large field and intervention studies, and improves resolution of species distribution within and between communities.

**Methods:**

This study reports differences in the prevalence of infections with all four human malarial species and of mixed infections as diagnosed by LM and post-PCR ligase detection reaction – fluorescent microsphere (LDR-FMA) assay in 15 villages in the central Sepik area of Papua New Guinea.

**Results:**

Significantly higher rates of infection by *P. falciparum, P. vivax, P. malariae *and *Plasmodium ovale *were observed in LDR-FMA compared to LM diagnosis (p < 0.001). Increases were particularly pronounced for *P. malariae *(3.9% vs 13.4%) and *P. ovale *(0.0% vs 4.8%). In contrast to LM diagnosis, which suggested a significant deficit of mixed species infections, a significant excess of mixed infections over expectation was detected by LDR-FMA (p < 0.001). Age of peak prevalence shifted to older age groups in LDR-FMA diagnosed infections for *P. falciparum *(LM: 7–9 yrs 47.5%, LDR-FMA: 10–19 yrs 74.2%) and *P. vivax *(LM: 4–6 yrs 24.2%, LDR-FMA: 7–9 yrs 50.9%) but not *P. malariae *infections (10–19 yrs, LM: 7.7% LDR-FMA: 21.6%). Significant geographical variation in prevalence was found for all species (except for LM-diagnosed *P. falciparum*), with the extent of this variation greater in LDR-FMA than LM diagnosed infections (overall, 84.4% vs. 37.6%). Insecticide-treated bednet (ITN) coverage was also the dominant factor linked to geographical differences in Plasmodium species infection prevalence explaining between 60.6% – 74.5% of this variation for LDR-FMA and 81.8% – 90.0% for LM (except *P. falciparum*), respectively.

**Conclusion:**

The present study demonstrates that application of molecular diagnosis reveals patterns of malaria risk that are significantly different from those obtained by standard LM. Results provide insight relevant to design of malaria control and eradication strategies.

## Introduction

Malaria is an infectious disease that shows considerable spatial heterogeneity on global [1,2], regional [3-6] and local scales [7-10]. While it has been possible at some levels of resolution to relate variation in prevalence or incidence of infections with differences in environment and measures of transmission potential (such as entomological inoculation rates) on global and regional levels [4,6,11], it has been difficult to evaluate *Plasmodium *species prevalence and determine the causes of the often pronounced local heterogeneity in malarial prevalence [12].

As all human *Plasmodium *species share both host and vector, mixed species infections are commonly observed. An extensive review of the literature on mixed species malaria infections by Ritchie [13] concluded that there are geographic differences in the way that human malaria species interact and that these interactions may even change from year to year for a given location. In selected field surveys from areas with a *Plasmodium falciparum*, *Plasmodium malariae *and *Plasmodium vivax *mix, a deficit of mixed infections has generally been recorded [14,15], suggesting heterologous suppression between those parasite species. In contrast, a surplus of mixed infections has been observed in areas with *P. falciparum*, *P. malariae *and *Plasmodium ovale *[14,15]. However, there is generally great variability in results between studies and it cannot be ruled out that observed fluctuations in the frequency of mixed infections may be due to limitations in the sensitivity of light microscopic (LM) species identification [16]. More recent studies using PCR techniques found much higher levels of mixed infections [17] and random distribution of different infections [18,19]. Earlier studies of mixed infections in mostly asymptomatic people during cross-sectional surveys has not provided conclusive evidence for interactions between the different *Plasmodium *species.

Previous studies on spatial patterns of malarial infections were based exclusively on light microscopy diagnosis (LM) of infection, which has a significantly lower sensitivity for detecting malarial infections compared to PCR-based diagnostic assays [17,20]. The more limited sensitivity is a particular problem in areas such as Papua New Guinea (PNG) where four human malaria species are endemic (*P. falciparum, P. vivax, P. malariae *and *P. ovale*), and mixed species infections are very common [21]. In these circumstances LM has been observed to be particularly poor in detecting the less common *P. malariae *and *P. ovale *infections [22] as well as for accurate diagnosis of mixed species infections [16].

The developments of a high-throughput post-PCR, LDR-FMA species typing assay [23] now makes feasible the routine molecular diagnosis of malaria infections in large field and intervention studies. This assay evaluates *P. falciparum, P. vivax, P. malariae *and *P. ovale *simultaneously and is performed in 96-well plate format to ensure efficient sample processing. Overall high specificity and sensitivity of the assay provides more accurate assessment of minority species in mixed infections. In addition, good correlations between parasite density and median fluorescence intensity confirmed that the assay is semi-quantitative [23,24]. In a recent comparison of malarial age group-based infection patterns detected by LM vs LDR-FMA methods in samples of over 1000 people (all ages), an increase in the prevalence of non-*falciparum *compared to *falciparum *infections, and a significant shift in distribution of infections to older age groups by LDR-FMA compared to LM diagnosis was observed [24]. Moreover, we found that adolescents and adults very commonly harbour sub-microscopic malarial infections. Similarly, in a cohort of children 5–14 years we found a significantly lower incidence of new *P. vivax *compared to *P. falciparum *blood stage infections when diagnosed by LM but identical rates when PCR diagnosis was used [25].

It is, therefore, becoming clear that increased application of PCR-diagnosis significantly alters the understanding of the epidemiology of different *Plasmodium *species. In order to further evaluate the effect of the increased sensitivity of detection on patterns of *Plasmodium *species infection, here LM and LDR-FMA diagnostic methods were compared in a series of 15 cross-sectional population surveys at five distinct locations in the central Sepik region of East Sepik province, PNG.

## Methods

### Study site and design

The selected study sites in the Maprik and Wosera-Gawi districts cover a combined area of over 160 km^2 ^that is characterized by low hills, plains and riverine plains with a wet tropical climate [26]. The natural vegetation is lowland hill forest that has mostly been replaced by re-growth following cultivation. Extensive grasslands are common on the riverine plans near the Sepik River. The Wosera study site described previously [26] is situated near the center of this expanded study region. In the early 1990's malaria was found to be holoendemic with a peak prevalence of 77% in children 5–9 years of age in the central Wosera area [26], with entomological inoculation rates (EIR) estimated at 35.4 infective bites/person-year for *P. falciparum*, 12.1 for *P. vivax*, and 9.6 for *P. malariae *[27]. By comparison, recent human studies in the Wosera found prevalence of *P. falciparum *to be significantly reduced [24].

**Figure 1:**
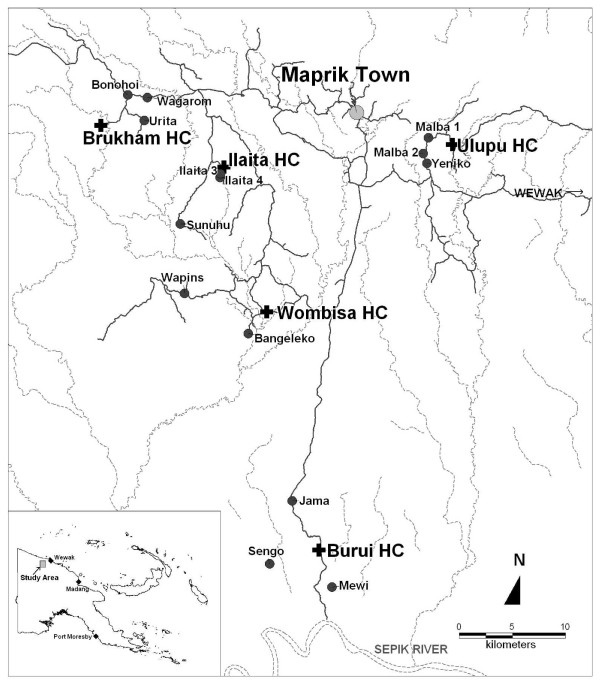
**Locations of cross-sectional surveys in vicinity of 5 health centres (HC) in the Middle Sepik Region of Papua New Guinea**. Wombisa village and health centre are in the same location.

For the present study five distinct areas surrounding local health facilities (i.e. Brukham, Burui, Ilaita, Ulupu and Wombisa HC) were selected (Figure 1). Brukham, Ilaita and Ulupu are situated in the foothills of the Prince Alexander range, Burui and Wombisa are in the Sepik River plain. In April and May 2005, 3 villages were surveyed in each of the five areas. In order to achieve a near-random sample of the village population a household-based sampling strategy was pursued. A number of households with a total population of 150–200 persons were included in each survey. The surveys included every member of the selected households who could be reached on the day of survey. Individual informed consent was obtained from all study participants (or consent granted by parents or guardians for inclusion of children) using study protocols evaluated and approved by the PNG Medical Research Advisory Committee (MRAC 00.26 & MRAC 05.20).

From each household a semi-structured questionnaire was administered to collect data on type of house, household assets, education of the parents, personal use of bednets, recent health facility attendance and use of antimalarial drugs. From each individual, a thick and thin film was prepared on a single slide and a 250 μl blood sample collected into a K+EDTA microtainer from a finger or heel prick. Haemoglobin measurements were made from this blood sample using a HemoCue 201+ Hb meter (Angholm, Sweden); remaining blood was preserved for extraction of DNA.

A socio-economic index was created using data on household ownership of consumer durables (i.e. bed, mattress, bednet, chairs, umbrella, clothing cupboard, kerosene pressure lamp, kerosene cooker, electric torch, radio, television, car). Households that owned < five consumer durables were classified as "low", > five consumer durables were classified as "medium", those with > 10 as "high" socio-economic status.

### Blood smear preparation and examination

All smears were stained with 2.5% buffered Giemsa (pH 7.2) for 35 minutes and examined by LM. Slides were declared negative if no parasites were seen in 100 thick film fields. The parasite species in positive films were identified and densities were recorded as the number of parasites/200 WBC. Densities were calculated assuming 8,000 WBC/μl [26]. Sexual and asexual stage parasite densities were reported separately for *P. falciparum *only. All slides with densities less than 200/μl, along with a randomly selected 20% of all blood films were routinely re-examined. If less than 80% concordance was achieved between LM evaluations, the entire batch of slides was re-read.

### DNA extraction and molecular diagnostic assay

Parasite DNA was extracted from the cell pellets using QIAmp 96 DNA Blood kits (Qiagen, CA).

A semi-quantitative post-PCR, ligase detection reaction/fluorescent microsphere assay (LDR-FMA) [23] was used to determine the presence of infection by *P. falciparum, P. vivax, P. malariae *and *P. ovale*. The design and sensitivity of this assay has been described previously [23,24,28]. In short, this assay combines PCR amplification of the 18S ribosomal RNA gene (491–500 bp fragments) using genus specific primers, followed by a multiplex species-specific ligation detection reaction (LDR). The LDR products are hybridized to Luminex^® ^FlexMAP™ "classification" bead sets (5'), and receive "reporter" labelling following incubation with streptavidin-R-phycoerythrin that binds to biotin (3'). Doubly labelled species-specific LDR complexes are detected using a Bio-Plex array reader (Bio-Rad Laboratories, Hercules, CA). Species-specific fluorescence data were collected by Bio-Rad software, Bio-Plex Manager 3.0 (Bio-Rad Laboratories, Hercules, CA). In order to guarantee maximum sensitivity for the detection of *Plasmodium *infections the PCR-cycle number was set at 35. Differentiation of negative from positive fluorescent signals was performed by comparing median fluorescent intensity (MFI) from study participants against values obtained from two negative controls on each 96-well plate. Cut-off values for positivity were set at the two standard deviations above the mean MFI of negative controls.

### Data analysis

Differences in prevalence of malarial infections among villages and areas and univariate association between categorical explanatory variables were tested using χ^2 ^test, multivariate predictors of infection risk were determined using logistic regression. Non-parametric Spearman's correlations were used to compare parasite density by light microscopy and mean fluorescence intensities, while parametric correlations were calculated for the association of prevalence of infection and mean haemoglobin at village level.

Within and between village variance in risk of malarial infections were estimated using a hierarchical Bayesian logistic model, i.e.

r_i _~ Bernoulli(p_i_)

where logit (p_i_) = β_a(i) _+ σ_j(i) _+ θ_j(i)k(i)_.

and "i" indexes the individuals, "a" the age-groups, "j" the area and "k" the village within area. "r_i_" indicates whether individual "i" is infected with a given *Plasmodium *species, "p_i_" denotes expected value, "β_a(i)_" is a fixed effect for the age group, "σ_j(i)_" is a random effect for the area and the "θ_j(i)k(i)_" is a random effect term for the village. In the models adjusting for bednet coverage, an additional linear effect is introduced into the model. All models were fitted using WinBugs 1.4 [29] assuming normal priors, with the distributions of the random effects centred on zero.

Variance components were estimated for all species with and without adjusting for bednet coverage. The relative contribution of between-area variance was determined by dividing the area variance component with the total spatially structure variance, i.e σ_j(i)_/(σ_j(i)+ _θ_j(i)k(i)_). The effect of difference in bednet coverage on spatially structured variance was determined by comparing the individual variance component from models with and without inclusion of the bednet covariate. Variance components, their relative sizes, and changes following adjustment for bednet coverage are all reported on a logit scale.

## Results

A total of 2,744 volunteers from 15 villages in five distinct geographical areas (Figure 1) participated in the cross-sectional surveys. Of these 121 (4.4%) were excluded because of lacking demographic or LM data, while insufficient finger-prick blood sample for LDR-FMA analysis led to exclusion of an additional 96 (3.5%) individuals. Overall 2,527 participants from 659 households with completed demographic information (median participation per household: 4, range (1–17)) were available for study. Of these 1,331 (52.7%) were female, 982 (38.9%) children < 10 yrs and 1,189 (47.1%) adults > = 20 yrs of age.

In this population, 1,121 (44.4%) and 1,844 (73.0%) participants had LM and LDR-FMA detectable *Plasmodium *species infections, respectively (Table 1). Diagnosis of infections by LM and LDR-FMA were highly significantly associated (X^2 ^> = 493.6, p < 0.001) for all species except *P. ovale *which is rarely detected by LM in PNG [22]. While the higher sensitivity of the LDR-FMA assay is reflected in a large number of infections that were only detected by this assay (*P. falciparum*: 667, *P. vivax *561, *P. malariae *251, *P. ovale *121), very few LM-positive infections were not detected by the LDR-FMA assay (*P. falciparum*: 46, *P. vivax *28, *P. malariae*: 12, *P. ovale*: 0). Fluorescence intensities of samples that were positive by both LM and LDR-FMA were significantly higher than those that were only LDR-FMA positive (*P. falciparum*:11,963 vs 4,970; *P. vivax*: 10,718 vs 5,109; *P. malariae*: 10,630 vs 6,422; all t-tests p < 0.001). For infections positive by both methods, fluorescence intensities were significantly correlated with densities by LM for *P. falciparum *(rho = 0.51, p < 0.001) and *P. vivax *(rho = 0.41, p < 0.001) but not for P. *malariae *(rho = -0.05, p = 0.67).

**Table 1:** Prevalence of malarial infections (as diagnosed by LM and LDR-FMA assay) in study communities.

			**Light microscope**					**LDR – FMA**				
				
**Area**	**Village**	**N**	**Pf**	**Pv**	**Pm**	**Po**	**All**	**Pf**	**Pv**	**Pm**	**Po**	**All**
			**%**	**%**	**%**	**%**	**%**	**%**	**%**	**%**	**%**	**%**
***Burui***		***489***	***26.8***	***9.0***	***1.8***	***0.0***	***36.1***	***43.4***	***28.4***	***4.7***	***3.1***	***63.2***
	Jama	203	22.2	8.9	1.0	0.0	32.0	35.0	27.6	2.5	3.4	59.1
	Sengo	153	29.4	7.2	3.3	0.0	38.6	49.7	22.9	6.5	3.9	62.8
	Mewi	133	30.8	11.3	1.5	0.0	41.4	48.9	36.1	6.0	1.5	69.9
												
***Wombisa***		***491***	***29.7***	***10.8***	***2.0***	***0.0***	***40.5***	***53.8***	***32.4***	***11.4***	***3.1***	***72.7***
	Wapins	145	21.4	11.0	2.8	0.0	34.5	49.7	29.0	8.3	4.1	69.7
	Bangeleko	172	33.7	6.4	1.2	0.0	39.5	58.7	32.6	14.5	1.2	76.7
	Wombisa	174	32.8	14.9	2.3	0.0	46.6	52.3	35.1	10.9	4.0	71.3
												
***Ulupu***		***521***	***28.4***	***17.1***	***2.5***	***0.0***	***43.7***	***52.2***	***39.9***	***10.0***	***4.8***	***72.6***
	Malba 1	163	23.3	14.7	0.6	0.0	36.8	50.3	43.6	9.2	3.1	76.1
	Malba 2	174	28.2	23.0	5.7	0.0	50.0	58.6	40.8	14.4	6.3	74.1
	Yeniko	184	33.2	13.6	1.1	0.0	44.0	47.8	35.9	6.5	4.9	67.9
												
***Brukham***		***515***	***35.0***	***15.9***	***4.5***	***0.0***	***47.8***	***60.8***	***39.8***	***18.1***	***6.6***	***77.3***
	Urita	166	28.9	18.7	3.6	0.0	41.6	60.8	45.2	17.5	6.6	78.3
	Bonohoi	174	32.8	8.6	4.6	0.0	41.4	54.6	33.9	16.1	6.3	71.8
	Wagarom	175	42.9	20.6	5.1	0.0	60.0	66.9	40.6	20.6	6.9	81.7
												
***Ilaita***		***511***	***30.0***	***19.6***	***8.6***	***0.0***	***52.6***	***63.8***	***37.2***	***22.3***	***6.3***	***78.7***
	Sunuhu	188	45.2	14.9	15.4	0.0	67.6	78.7	38.8	30.9	9.0	85.7
	Ilaita 3	148	15.5	18.9	4.1	0.0	36.5	56.1	35.8	14.2	3.4	75.7
	Ilaita 4	175	24.6	25.1	5.1	0.0	50.3	54.3	36.6	20.0	5.7	73.7
												
**Overall**		**2,527**	**29.90**	**14.60**	**3.90**	**0.00**	**44.4**	**54.90**	**35.70**	**13.40**	**4.80**	**4.80**

Pf = *P. falciparum*, Pv = *P. vivax*, Pm = *P. malariae*, Po = *P. ovale*

**Figure 2:**
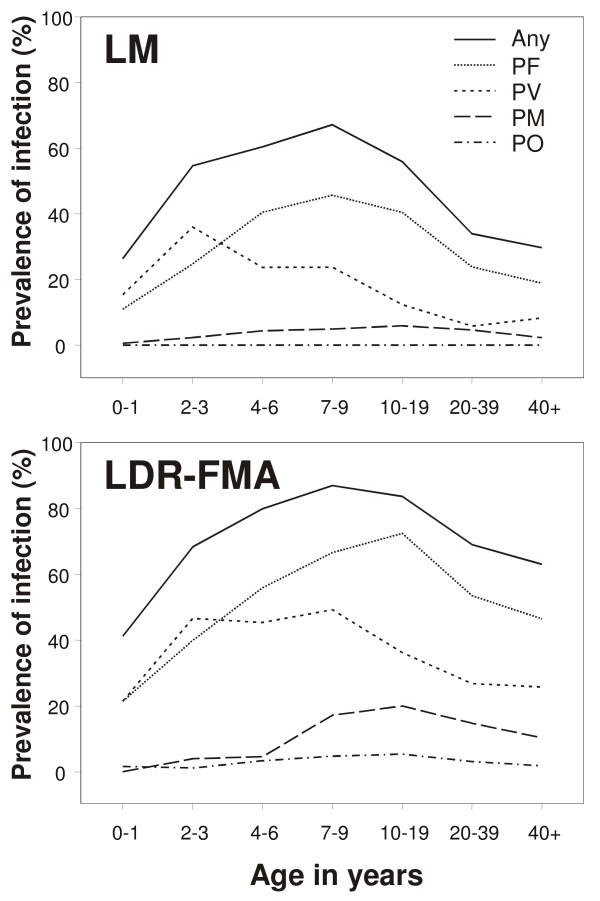
**Age specific prevalence rates of malarial infections as diagnoses by light microscopy (LM) and LDR-FMA**.

By LM, *P. falciparum *was the most common parasite with a prevalence of 29.9% (756/2,527) followed by *P. vivax *and *P. malariae *with a prevalence of 14.6% (368/2,527) and 3.9% (99/2,527), respectively (Table 1). No infections with *P. ovale *were detected by LM. *Plasmodium *infections were most frequent in the 7–9, and 4–6 year-old children (67.2%, CL_95_: 61.2–72.8 and; 60.4%, CL_95_: 54.9–65.8) respectively, followed by adolescents (10–19 years: 55.9%, CL_95_: 50.6–61.1), 2–3 year olds (54.7%, CL_95_: 47.7–61.5), adults (20–39 years: 33.9%, CL_95_: 30.5–37.4, > = 40 years: 29.7, CL_95_: 25.5–34.3)) and infants (0–1 years: 26.4%, CL_95_: 20.1–33.4). *P. vivax *reached peak prevalence in younger children (Figure 2: 4–6 years: 24.2%, C_95_: 22.1–26.3) than *P. falciparum *(7–9 years: 47.5%, CL_95_: 41.1–53.7) or *P. malariae *(10–19 years: 7.7%, CL_95_: 3.7–8.8).

A significantly higher proportion of individuals were positive for infection when diagnosed by LDR-FMA (Table 1). This was the case for all species but was most pronounced for the less common species; *P. falciparum *prevalence increased 1.8 fold (29.9% to 54.9%, p < 0.001), *P. vivax *2.4-fold (14.6% to 35.7%, p < 0.001) and *P. malariae *3.4-fold (3.9% to 13.4%, p < 0.001), while *P. ovale *was detected in samples from 4.8% (p < 0.001) of all participants. In parallel with these increases from LM to LDR-FMA, more mixed infections were detected (Table 2, 4.0% to 28.6%, p < 0.001) and mixed infections were detected in a greater proportion of the infected samples (9.0% to 39.2%, p < 0.001).

**Table 2:** Comparison of observed and expected *Plasmodium *species assemblages by different diagnostic techniques.

	**Light microscopy**			**LDR-FMA**		
		
	**Observed**	**Expected^1^**	**X^2^**	**Observed**	**Expected**	**X^2^**
neg	1406	1453.2	1.5	683	605.0	10.1
						
Pf	660	620.4	2.5	704	736.1	1.4
Pv	276	247.7	3.2	350	335.2	0.6
Pm	84	59.3	10.3	45	93.4	25.1
Po	0	0.6	0.6	22	30.4	2.3
						
Pf+Pv	86	105.7	3.7	363	407.9	4.9
Pf+Pm	9	25.3	10.5	136	113.7	4.4
Pf+Po	0	0.2	0.2	27	37.0	2.7
Pv+Pm	5	10.1	2.6	26	51.8	12.8
Pv+Po	0	0.1	0.1	7	16.9	5.8
Pm+Po	0	0.0	0.0	1	4.7	2.9
						
Pf+Pv+Pm	1	4.3	2.5	99	63.0	20.6
Pf+Pv+Po	0	0.0	0.0	33	20.5	7.6
Pf+Pm+Po	0	0.0	0.0	8	5.7	0.9
Pv+Pm+Po	0	0.0	0.0	6	2.6	4.4
Pf+Pv+Pm+Po	0	0.0	0.0	17	3.2	60.4
						
		Total:	37.9		Total:	167.0
			p < 0.001			p < 0.001

^1 ^Expected values calculated under multiple kind lottery model

^2 ^X^2 ^calculated as (Observed-Expected)^2^/Expected

Pf = *P. falciparum*, Pv = *P. vivax*, Pm = *P. malariae*, Po = *P. ovale*

As in earlier studies [18,19,24], we used the multiple-kind lottery model to determine whether there was a random distribution of *Plasmodium *species in the survey population (Table 2). Among both LM and LDR-FMA diagnosed infections a significant departure from random distribution was observed, albeit in different ways. By LM diagnosis single species infections were observed at significantly higher frequencies than expected, and different mixed *Plasmodium *species infections were observed at frequencies that were equal to, or lower than the expected frequencies. Overall, mixed infections were found in only 4.0% of all samples compared with the expected 5.8% (p < 0.001). Fewer mixed species infections were observed in Burui and Wombisa compared to the other areas (Additional files 1 &2). In a given individual, infection with one species was negatively associated with the presence of any other species (Table 3). By LDR-FMA diagnosis, single and double species infections were observed at frequencies equal to or less than expected, whereas triple and quadruple infection assemblages (Table 1: 6.5% vs 3.8%, p < 0.001) and negative samples (27.0% vs 23.9%, p = 0.002) were significantly more common than expected. Among LDR-FMA diagnosed infections, however, positive associations were observed for all species pairs except *P. falciparum*/*P. vivax *(Table 3) in any given individual. While the general trend of negative associations between species in LM, and positive associations in LDR-FMA diagnoses are consistent across different age groups, the effects tended to be more pronounced in adults and adolescents (> 10 yrs) compared to children, except for LDR-FMA diagnosed *P. falciparum*, *P. malariae *and *P. ovale *which show a stronger association in children (Table 3).

**Table 3:** Associations between infections with different *Plasmodium *species as observed by different diagnostic techniques.

	**Light microscopy**			**LDR – FMA**		
		
	**OR^1^**	**CL_95_**	**P-value**	**OR^1^**	**CL_95_**	**P-value**
**Overall**						
Pf vs Pv	0.69	[0.53,0.90]	0.004	1.12	[0.96,1.34]	0.145
Pf vs Pm	0.25	[0.11,0.49]	< 0.001	3.14	[2.39,4.15]	< 0.001
Pf vs Po	--			2.00	[1.33,3.07]	< 0.001
Pv vs Pm	0.37	[0.13,0.84]	0.014	1.49	[1.17,1.89]	< 0.001
Pv vs Po	--			2.00	[1.39,2.98]	< 0.001
Pm vs Po	--			2.47	[1.56,3.81]	< 0.001
						
**Children < 10 yrs**						
Pf vs Pv	0.71	[0.52,0.97]	0.034	1.04	[0.81,1.34]	0.761
Pf vs Pm	0.41	[0.17,1.00]	0.049	6.78	[3.63,12.67]	< 0.001
Pf vs Po	--			2.50	[1.29,4.82]	0.006
Pv vs Pm	0.41	[0.14,1.17]	0.096	1.09	[0.70,1.70]	0.713
Pv vs Po	--			1.97	[1.07,3.63]	0.029
Pm vs Po	--			6.19	[3.18,12.05]	< 0.001
						
**Adolescents & adults > = 10 yrs**						
Pf vs Pv	0.38	[0.22,0.65]	< 0.001	1.30	[1.04,1.63]	0.020
Pf vs Pm	0.17	[0.06,0.46]	0.001	2.37	[1.76,3.21]	< 0.001
Pf vs Po	--			1.74	[1.06,2.87]	0.030
Pv vs Pm	0.35	[0.08,1.44]	0.145	1.96	[1.49,2.59]	< 0.001
Pv vs Po	--			2.15	[1.35,3.42]	0.001
Pm vs Po	--			1.51	[0.66,2.62]	0.150

^1 ^OR: Odd-ratio. OR < 1 indicated negative, OR > 1 a positive association, CL_95_: 95% confidence limit

Pf = *P. falciparum*, Pv = *P. vivax*, Pm = *P. malariae*, Po = *P. ovale*

For all *Plasmodium *species, the increase in apparent prevalence of infections detected by the LDR-FMA assay was proportionally larger in adults than in children (Figure 2). As a consequence the prevalence of infection appeared to peak at a later age for LDR-FMA-detectable infections than when diagnosis was performed by LM (Figure 2). Prevalence of infections peaked in adolescents (10–19 years: *P. falciparum*: 74.2%, CL_95_: 69.3–78.6; *P. malariae*: 21.6%, CL_95_: 17.5–26.3; *P. ovale*: 7.0%, CL_95_: 4.6–10.2; mixed infections: 43.0%, CL_95_: 37.9–48.3) in all species except *P. vivax*, where infections were most commonly found in children 7–9 years of age (50.9%, CL_95_: 44.8–57.1).

The risk of infections with malaria parasites was independently associated with a number of risk factors. The most important predictors were coverage of insecticide treated ITNs, educational level of the female household head and socio-economic status of family (Table 4). ITN coverage was significantly associated with a reduction in risk of infection with all different malaria species both by LM and LDR-FMA, although for *P. vivax *a decrease was only observed at high ITN coverage (> 90%). People living in households where the mother spoke English or had attended at least 3 years of schooling also had a significantly lower risk of being infected with *P. falciparum*, *P. vivax *(LDR-FMA diagnosed only) and *P. malariae *infections but not with *P. ovale*. Only risk of infection with *P. falciparum *was significantly reduced in households of higher socio-economic status, while difference in house construction was only associated with difference in risk of LDR-FMA diagnosed *P. falciparum *and *P. malariae *infection.

**Table 4:** Multivariate predictors of malarial infections in study populations.

		**Light microscopy**				**LDR – FMA**				
			
		**Pf**	**Pv**	**Pm**	**All**	**Pf**	**Pv**	**Pm**	**Po**	**All**
Bednet coverage^1^	AOR	0.90***		0.62***	0.78***	0.79***		0.69***	0.80***	0.80***
	CL_95_	[0.85,0.95]		[0.55,0.70]	[0.74,0.82]	[0.74,0.83]		[0.64,0.74]	[0.72,0.90]	[0.74,0.85]
Bednet coverage > 90%	AOR		0.43***				0.63***			
	CL_95_		[0.34,0.56]				[0.53,0.74]			
Mother speaks English	AOR	0.56***			0.77*	0.61***		0.50**		
	CL_95_	[0.41,0.76]			[0.59,0.99]	[0.47,0.7]		[0.41,0.93]		
Mother > 6 yrs education	AOR						0.72*			0.64**
	CL_95_						[0.53,0.99]			[0.46,0.88]
Economic Status										
Medium	AOR	0.74*								
	CL_95_	[0.58,0.93]								
High	AOR	0.59***			0.82*	0.75**				0.77**
	CL_95_	[0.46,0.75]			[0.69,0.98]	[0.47,0.79]				[0.64,0.93]
No windows	AOR					1.36*				
	CL_95_					[1.04,1.78]				
Screened windows	AOR							0.33***		
	CL_95_							[0.17,0.67]		
Walls from Sago palm	AOR							0.46*		
	CL_95_							[0.24,0.89]		
Modern toilet^2^	AOR									0.66*
	CL_95_									[0.45,0.97]

^1 ^Effect associated with a 10% increase in net use among all participants in study village

^2 ^Flush toilet or pit latrine with cement floor

AOR: Age-adjusted odd-ratio, CL_95_: 95% confidence limit. *** p < 0.001, ** p < 0.01, * p < 0.05.

For all species and both diagnostic methods, there were significant differences in prevalence of *Plasmodium *species infections among villages (Table 1, p < 0.001 for all species, except *P. ovale *(LDR-FMA) p = 0.03), and areas (p < 0.001 for all species, except *P. falciparum *(LM) p = 0.06) and *P. ovale *(LDR-FMA) p = 0.01). By LM, infection prevalence for individual villages varied between 15.5% to 45.2% for *P. falciparum *(coefficient of variance (CV): 26.4%), 6.4% to 25.1% for *P. vivax *(CV: 39.8%), 0.6% to 15.4% for *P. malariae *(CV: 94.5%) and 32.0% to 67.6% for any *Plasmodium *infections (CV: 21.9%). *P. vivax *and *P. malariae *infections were found more commonly in the villages in the foot hills (Ulupu (*P. vivax *only), Brukham & Ilaita) compared to the villages situated on the Sepik River flood plain. The prevalence of LDR-FMA detectable infections were significantly associated with those by LM (*P. falciparum*: r^2 ^= 0.65, p = 0.008, *P. vivax*: r^2 ^= 0.67, p = 0.007, *P. malariae*: r^2 ^= 0.85, p < 0.001). Although variation in infection prevalence between villages was less pronounced (*P. falciparum*: 35.0% to 78.7%, CV: 17.8%); *P. vivax*: 22.9% to 45.2%, CV: 16.8%; *P. malariae*: 2.5% to 30.9%, CV: 55.3%); *P. ovale*: 1.2% to 9.0%, CV: 45.3% and any infection: 59.1% to 85.6%, CV: 17.8%), broadly similar geographical trends were observed. Interestingly, prevalence of any malaria infection by both LM and LDR-FMA was significantly associated with a lower population mean Hb level (LM: r^2 ^= -0.69, p = 0.004; LDR-FMA: r^2 ^= -0.75, p = 0.001) and a higher incidence of moderate-to-severe anaemia (Hb < 8 g/dl, LM: r^2 ^= 0.61, p = 0.016; LDR-FMA: r^2 ^= 0.65, p = 0.009). When diagnosed by LM, the reductions in age- and gender-adjusted Hb levels were most strongly associated with the presence of a mixed infection (-1.2 g/dl, CL_95_: 0.9–1.4, p < 0.001), followed by *P. malariae *(-0.6 g/dl, CL_95_: 0.3–0.9, p < 0.001), *P. falciparum *(-0.5 g/dl, CL_95_: 0.4–0.7, p < 0.001) and *P. vivax *single infections (-0.3 g/dl, CL_95_: 0.1–0.5, p = 0.005). Among LDR-FMA diagnosed infections only mixed species (-0.4 g/dl, CL_95_: 0.2–0.5, p < 0.001) and *P. falciparum *single infections (-0.5 g/dl, CL_95_: 0.3–0.7, p < 0.001) were associated with a significant reduction in Hb levels. Hb reductions were larger in children than adults and adolescents.

The Bayesian estimation of among-area and village-within-area variance components revealed differences in scale of this geographical variation in risk of infection among the different *Plasmodium *species and between methods of diagnosis (Table 5). Among LM diagnosed infection, area-specific differences accounted for only 11.5% of variation in rates of infection with *P. falciparum*, but for 54.9% and 53.7% of variation in rates of *P. vivax *and *P. malariae *infections respectively. With LDR-FMA the proportion of location-structured variation increased to 53.8% for *P. falciparum*, 88.1% for *P. vivax *and 83.7% for *P. malariae*, 86.4% of variation in rates of *P. ovale *infections was observed between sites.

**Table 5:** Posterior medians of among-area and village-within-area variance components.

	**Pf**	**Pv**	**Pm**	**All**	**Pf**	**Pv**	**Pm**	**Po**	**All**
**Age adjusted**									
Areas	0.023	0.259	0.554	0.125	0.180	0.108	0.726	0.221	0.233
Villages within area	0.174	0.231	0.503	0.213	0.165	0.018	0.149	0.046	0.050
% between-area^1^	11.5%	54.9%	53.7%	37.6%	53.8%	88.1%	83.6%	86.4%	84.4%
**Age + bednet adjusted**									
Areas	0.031	0.026	0.060	0.023	0.048	0.042	0.225	0.060	0.059
Villages within area	0.163	0.175	0.102	0.133	0.091	0.013	0.033	0.017	0.015
% between-area^1^	15.9%	12.6%	41.2%	14.7%	34.0%	80.2%	89.9%	80.1%	82.6%
**Relative change^2^**									
Areas	+36.6%	-90.0%	-89.2%	-81.8%	-73.4%	-60.6%	-69.0%	-73.0%	-74.5%
Villages within area	-6.7%	-24.4%	-79.6%	-37.8%	-44.6%	-29.5%	-78.2%	-61.7%	-69.4%
Total spatial variance^3^	-1.6%	-59.1%	-84.6%	-54.1%	-59.7%	-56.2%	-70.6%	-71.1%	-73.6%

^1 ^Proportion of among-area variance of total spatial variance measured on a logit scale (i.e. σ_j(i)_/(σ_j(i)+ _θ_j(i)k(i)_, see methods). Posterior median estimated from model does not exactly correspond proportion calculated from posterior medians of individual variances.

^2 ^Relative chance of posterior median of variance components from bed-net adjusted to non-adjusted model.

^3 ^Sum of posterior medians of among-area and village-within-area variance components (i.e. σ_j(i)+ _θ_j(i)k(i)_)

Pf = *P. falciparum*, Pv = *P. vivax*, Pm = *P. malariae*, Po = *P. ovale*

**Figure 3:**
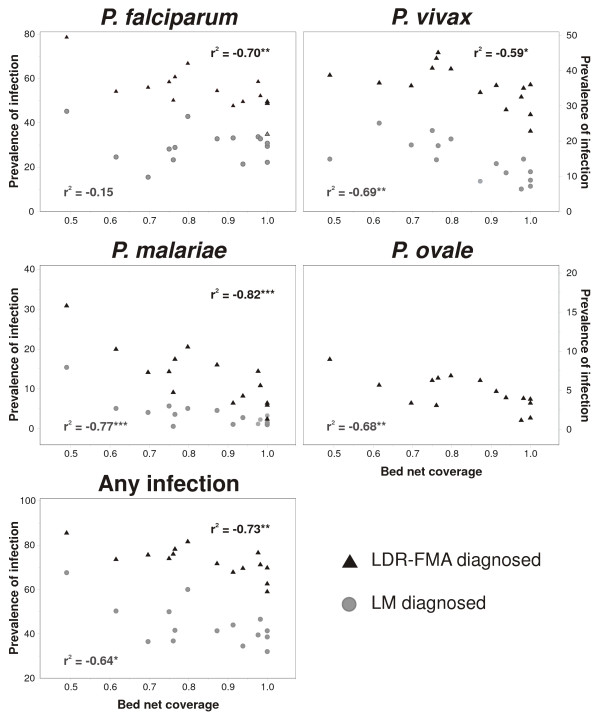
**Association of bednet coverage and parasite prevalence rates in 15 study villages**. *** p < 0.001, ** p < 0.01, * p < 0.05.

The prevalence of *Plasmodium *species infections was significantly reduced in villages with higher ITN coverage (Figure 3). Such a negative association was observed for all species when diagnosed by LDR-FMA (r^2^: -0.59 to -0.82, p < 0.05) as well as *P. vivax *(r^2^: -0.69, p < 0.01) and *P. malariae *(r^2^: -0.77, p < 0.001) but not *P. falciparum *(r^2^: -0.15) when diagnosed by LM. Accounting for differences in bednet coverage among villages consequently reduced total spatial variance by 56–85% for all species and both diagnostic methods, except for LM-diagnosed *P. falciparum *infections (-2%). In contrast, substantial differences were observed between the two diagnostic methods and between *Plasmodium *species in regard to the spatial level at which the effect of bednet coverage was most pronounced (Table 5). Among LDR-FMA diagnosed infections, bednet coverage was associated with a comparable reduction in among-area and village-within-area variances of *P. malariae *and *P. ovale *prevalence rates, but a stronger reduction in among-area variance was observed for *P. falciparum *and *P. vivax*. Among LM diagnosed *P. malariae *infections, reduction in among-area variance were comparable to those observed at village-level, while the increased reduction in among-area variance of *P. vivax *infection was even more pronounced than among LDR-FMA diagnosed infection. Adjusting for bednet coverage further increased the differences in the proportion of spatial variance observed at among-area level variance observed in LM (13–41%) compared to LDR-FMA diagnosed infection (34–90%).

## Discussion

With over 2,500 samples processed, the present study is the largest epidemiological study to-date that has used a post-PCR LDR-FMA assay for the diagnosis of malarial infection on all samples collected. The direct comparison of LM and LDR-FMA results confirmed that the LDR-FMA assay is both significantly more sensitive than LM and semi-quantitative [23,24], with the increased sensitivity most notable for *P. malariae *and *P. ovale *infections.

The higher sensitivity of the LDR-FMA in detecting low density infection was most notable in the assessment of mixed species assemblages and in age-specific prevalence. In concordance with the observations by Kasehagen *et al*. [24], a significant deficit of mixed species infections was observed in LM diagnosis, but an excess of high-level mixed infections, and interestingly also of uninfected study participants, were detected by LDR-FMA diagnosis. Prevalence of infections detected by LDR-FMA peaked at a later age than did infections diagnosed by LM (Figure 2), confirming that adolescents and adults contribute significantly more to the total burden of infections in a population than appreciated when only LM diagnosis is used [24]. Having observed the same patterns of infections in two independent surveys suggests that similar changes may be generally observed when PCR-based diagnosis is used in epidemiological studies.

Associations between different *Plasmodium *species have long been a subject of investigation, but results have been inconsistent and inconclusive (see the review by Richie [13]). Since the vectors of different Plasmodia are the same, it may be expected that the different parasites would be associated, unless there is immune-mediated competition, species-specific difference in red blood cell susceptibility. Meta-analysis of 19 studies by McKenzie and Bossert [15] found a tendency for statistically significant surplus of mixed infections by *P. falciparum-P. malariae-P. ovale *and for deficits in *P. falciparum-P. malariae-P. vivax*. The present analyses suggest that these deficits may be artifacts of the lower sensitivity of LM, since they were seen in the PNG data only when the assessment was made by LM. This is further highlighted by the weaker negative association in children under 10 that have a lower immune status and thus a more limited control of parasitaemia [30]. A recent series of cross-sectional studies in Malawi [31] that used PCR for diagnosis also found an significant increase of mixed infection that we more pronounced in adults than children. These observations are consistent with a suggestion [13] that parasite densities may be suppressed by co-infections, but that co-infections do not prevent blood stage infections, or stimulate cross-species immunity capable of limiting infections.

With both diagnostic methods considerable variation in prevalence of infections was observed among villages for all *Plasmodium *species. While prevalence by LM and LDR-FMA was strongly correlated and overall geographical patterns comparable, LM-based prevalence showed a much higher degree of variability within individual areas for all *Plasmodium *species than LDR-FMA-based prevalence. In particular, for the non-*falciparum *species, almost all spatial variation in LDR-FMA-based rates of infection was observed as difference between areas. The proportionally higher within area variance is likely to be related to the lower sensitivity of LM (both for low-level infection and correct identification of infecting species). Differences in the quality of different slide batches, or between-reader variation may further contribute to within-area variation. In intervention trials where more than one slide is made and all slides have at least two independent reads these types of variability may be reduced. In large scale epidemiology studies or in monitoring of malaria interventions where thousands of people are screened, this is not necessarily common practice. If the trend for a decrease in survey-to-survey variation in PCR-based diagnosis holds true elsewhere, the heterogeneity of malaria risk at the local level may have been overestimated in earlier studies. Results of this nature would emphasize the importance of implementing molecular diagnostic methods.

Interestingly, the least amount of between area variation (by both LM and LDR-FMA) was observed in the most common parasites, i.e. *P. falciparum*. Whether this reflects a true difference in ecology of different *Plasmodium *species in the study area is difficult to determine from a single cross-sectional survey. In addition, multiple *P. falciparum *strain infections are commonly observed in PNG [32] and the true amount of geographical difference in transmission may be obscured if only presence or absence, but not the number of infecting strains were detected.

The coverage of ITNs in a given village was the best predictor of risk of malaria infections at individual and village levels. Both high coverage of ITNs in the village of residence as well as personal ITN use significantly reduced a participant's risk of being infected with any of the human malaria species. Interestingly, personal use was less strongly associated with protection (data not shown) than overall coverage in a given village. This suggests that if high coverage is achieved, ITN may have an effect on overall transmission and thereby also protect people who do not always sleep under a ITN [33]. The protective effect of ITNs against *P. falciparium *is well described [34] and there increasing evidence for their effectiveness against *P. vivax*, although the effect may be smaller than that against *P. falciparum *[35-37]. To date there has been very little information on the effect of ITNs on *P. malariae *and *P. ovale*. While ITNs were highly effect in reducing *P. malariae *in a study in Burkina Faso[38], this may be the first description of an effect of ITNs on risk of *P. ovale *infections.

Differences in ITN coverage among villages also reduced total spatial variance of Plasmodium infections (measured on the logistic scale) by up to 85%. Similar spatial associations of coverage in untreated bednets with reduced (peak) prevalence and increased age at peak prevalence were observed *P. falciparum*, *P. vivax *and *P. malariae *in earlier studies in the Wosera [27]. In addition, high coverage of untreated bednets led to significant reduction in sporozoite rates in mosquito vectors [39]. In African studies high ITN coverage reduced *P. falciparum *transmission by up to 90% [11,40]. Therefore, it is very likely that if sufficiently high coverage rates can be achieved, the ongoing PNG national bednet distribution program will not only reduce the burden of illness, but also have an impact of prevalence of infections. The very high reductions in between-area variation in *P. malariae *and *P. ovale *prevalence suggest that ITNs may be quite effective for the control of these species, while control of *P. vivax *may be more difficult as indicated by the smallest reduction in spatial variation (by LDR-FMA). Interestingly, the variation in prevalence of infection was closely linked to a difference in mean haemoglobin levels in the study villages, indicating that control of malaria through ITNs may also result in improved haemoglobin levels. Given the well known problems with spurious correlations in ecological comparisons, these associations with ITN coverage need to be interpreted with care. Direct evaluation of the ongoing PNG national bednet distribution program on the transmission of non-falciparum species is needed.

The only other factor consistently associated with a reduced prevalence of *Plasmodium *infections is the level of maternal education. It is well know that educated mothers have a better understanding of health, seek treatment more promptly, and use ITN more regularly [41-43] than uneducated mothers [44,45]. Similarly, higher socioeconomic status is generally associated with better health outcomes. Interestingly, there was no consistent association of house-type with prevalence of malaria infections of any species. This suggests that different housing types are similarly attractive to the local mosquito vector species. The tendency of many PNG anophelines to bite outdoors readily in the early or late hours of the night [46] may further reduce any effect of different house types on risk of *Plasmodium *species infection.

The present study demonstrates that application of the high-throughput, PCR-based LDR-FMA diagnostic assay is feasible in large malaria field studies. Additionally, results show that epidemiological patterns of malaria risk detected by molecular diagnosis differ significantly from risk assessed by LM. Overall, results from this study enhance our understanding of the impact that low level asymptomatic infection, infection in older age groups, and infection by less common malaria parasites contributes to the burden and transmission of *Plasmodium *species infections. In combination with high-throughput *Plasmodium *strain typing assays [47,48] and novel approaches to define the levels of gametocytemia[49], the accurate classification of *Plasmodium *infections in the field and the understanding of malaria epidemiology is significantly improved., Future large scale malaria field studies and, in particular, intervention trials would therefore benefit significantly by improving capacity for molecular diagnosis of *Plasmodium *infections. Increased investment into establishing molecular diagnosis capacity in malaria endemic countries should be considered a high priority.

## Competing interests

The authors declare that they have no competing interests.

## Authors' contributions

IM designed the study, analysed the data and wrote the first draft. SW, DM, SM, BK AS conducted the field work and read blood slides. SW and DM also assisted in data analyses. DTM and PAZ conducted LDR-FMA typing of all samples. TAS assisted with the spatial data analyses. All authors contributed to the interpretation of the data and the writing of manuscript.

## Supplementary Material

Additional file 1 **Table S1.** Comparison of observed species mixtures in different regions by LDR-FMA. Click here for file

Additional file 2 **Table S2. **Comparison of observed species mixtures in different regions by light microscopy. Click here for file
