# Supplementary material for: High sensitivity detection of Plasmodium species reveals positive correlations between infections of different species, shifts in age distribution and reduced local variation in Papua New Guinea
Source: Malar J. 2009 Mar 11;8:41. doi: 10.1186/1475-2875-8-41 (PMC2657150; doi:10.1186/1475-2875-8-41)
Supplement: Additional file 1 — Table S1. Comparison of observed species mixtures in different regions by LDR-FMA. [file 1475-2875-8-41-S1.pdf]

Additional File 1: Comparison of observed species mixtures in different regions by LDR-FMA.

	**Burui** n = 489	**Wombisa** n = 491	**Ulupu** n =521	**Brukham** n = 515	**Ilaita** n = 511
neg	180	134	143	117	109
Pf	144	153	133	137	137
Pv	82	72	81	63	52
Pm	6	9	9	7	14
Po	7	1	7	4	3
Pf+Pv	46	62	89	84	82
Pf+Pm	8	28	16	33	51
Pf+Po	4	7	5	9	2
Pv+Pm	2	8	5	6	5
Pv+Po	0	2	3	1	1
Pm+Po	0	0	0	1	0
Pf+Pv+Pm	6	10	20	34	29
Pf+Pv+Po	3	4	8	7	11
Pf+Pm+Po	1	0	0	2	5
Pv+Pm+Po	0	1	1	3	1
Pf+Pv+Pm+Po	0	0	1	7	9

Pf = *P. falciparum*, Pv = *P. vivax*, Pm = *P. malariae*, Po = *P. ovale*
