# Supplementary material for: High sensitivity detection of Plasmodium species reveals positive correlations between infections of different species, shifts in age distribution and reduced local variation in Papua New Guinea
Source: Malar J. 2009 Mar 11;8:41. doi: 10.1186/1475-2875-8-41 (PMC2657150; doi:10.1186/1475-2875-8-41)
Supplement: Additional file 2 — Table S2. Comparison of observed species mixtures in different regions by light microscopy. [file 1475-2875-8-41-S2.pdf]

Additional File 2: Comparison of observed species mixtures in different regions by light microscopy.

	**Burui** n = 489	**Wombisa** n = 491	**Ulupu** n =521	**Brukham** n = 515	**Ilaita** n = 511
neg	310	292	293	269	242
Pf	126	136	127	145	126
Pv	40	44	68	50	74
Pm	8	9	11	13	43
Po	0	0	0	0	0
Pf+Pv	4	9	20	28	25
Pf+Pm	1	1	1	6	0
Pf+Po	0	0	0	0	0
Pv+Pm	0	0	1	3	1
Pv+Po	0	0	0	0	0
Pm+Po	0	0	0	0	0
Pf+Pv+Pm	0	0	0	1	0
Pf+Pv+Po	0	0	0	0	0
Pf+Pm+Po	0	0	0	0	0
Pv+Pm+Po	0	0	0	0	0
Pf+Pv+Pm+Po	0	0	0	0	0

Pf = *P. falciparum*, Pv = *P. vivax*, Pm = *P. malariae*, Po = *P. ovale*
